# Past 200 kyr hydroclimate variability in the western Mediterranean and its connection to the African Humid Periods

**DOI:** 10.1038/s41598-022-12047-1

**Published:** 2022-05-31

**Authors:** Jon Camuera, María J. Ramos-Román, Gonzalo Jiménez-Moreno, Antonio García-Alix, Liisa Ilvonen, Leena Ruha, Graciela Gil-Romera, Penélope González-Sampériz, Heikki Seppä

**Affiliations:** 1 Department of Geography and Geosciences, Faculty of Science, University of Helsinki, Helsinki, Finland; 2 Andalusian Earth Sciences Institute (IACT, CSIC-UGR), Armilla, Granada, Spain; 3 Department of Stratigraphy and Paleontology, Faculty of Science, University of Granada, Granada, Spain; 4 Research Centre for Ecological Change, Organismal and Evolutionary Biology Research Programme, Faculty of Biological and Environmental Sciences, University of Helsinki, Helsinki, Finland; 5 Natural Resources Institute Finland, Oulu, Finland; 6 Research Unit of Mathematical Sciences, University of Oulu, Oulu, Finland; 7 Pyrenean Institute of Ecology (IPE-CSIC), Zaragoza, Spain; 8 Department of Ecology, Faculty of Biology, Philipps-Marburg University, Marburg, Germany

**Keywords:** Climate sciences, Environmental sciences

## Abstract

The Iberian Peninsula is located at the intersection between the subtropical and temperate climate zones and the paleoclimate records from this region are key to elucidate the varying humidity and changing dominance of atmospheric circulation patterns in the Mediterranean-North African region in the past. Here we present a quantitative hydroclimate reconstruction for the last ca. 200 kyr from southern Iberian Peninsula based on pollen data from the Padul lake sediment record. We use the newly developed Scale-normalized Significant Zero crossing (SnSiZer) method to detect not only the statistically significant precipitation changes but also to estimate the relative magnitude of these oscillations in our reconstruction. We identify six statistically significant main humid phases, termed West Mediterranean Humid Periods (WMHP 1–6). These humid periods correlate with other West/Central Mediterranean paleohydrological records, suggesting that similar climatic factors affected different areas of the Mediterranean. In addition, the WMPHs are roughly coeval with the African Humid Periods (AHPs) during high seasonality, suggesting the same North Atlantic ocean-atmospheric dynamics and orbital forcing as main drivers of both areas. In contrast, during low seasonality periods, the West Mediterranean still appears to be affected by the westerlies and the local Mediterranean rainfall systems with moderate-to-high precipitation, whereas West Africa was characterized by droughts.

## Introduction

Climate of the Iberian Peninsula in the western Mediterranean is characterized by high diversity, with a gradient from oceanic climate on the Atlantic coast to hot semi-arid climate in the south-southeast. This climatic diversity is reflected in the vegetation patterns, ranging from temperate forests to semi-deserts. Past changes in climate, such as those documented during the Holocene and last glacial period in the Sahara and North Africa^1–3^ and in northern Europe and the North Atlantic^4–7^, had a profound impact on the vegetation and environments in the Iberian Peninsula^8,9^, making it a key region for understanding paleoclimate changes. In addition, the comparison of paleoclimate records from the Mediterranean and North Africa is essential for understanding the climate mechanisms affecting both areas and the possible influence of the African monsoon on the Mediterranean climate. Several paleoclimate records have been published focusing on changes in temperature, moisture variability and paleohydrological conditions during the last interglacial-glacial cycle in the Mediterranean region based on pollen and dust records from marine and continental cores^10–12^ and isotope data from speleothems^13,14^, among others. In this respect, the Padul wetland is a unique Mediterranean site for paleoclimate studies as it has recorded the paleoenvironmental conditions of the last 1 Ma^15,16^. Our sediment core from Padul is one of the oldest continuous continental records in the Mediterranean region, spanning from the beginning of the penultimate glacial period until the present (the last ca. 200 kyr), making it a suitable and unique record for quantitative paleoclimate reconstructions. Moreover, its geographical location, being the southernmost continental archive in the Iberian Peninsula covering this period, makes it unique to explore paleoclimatological patterns implying the North Atlantic Oscillation (NAO) as well as North African climate variability.

The western Mediterranean is (was) affected by precipitation changes partially controlled by the NAO (NAO-like dynamics in the past), which mostly determine the vegetation dynamics and composition^17–19^. In the Mediterranean ecosystems, the vegetation is well-adapted to summer-droughts, whereas winter precipitation is critical for forest growth and controls regional forest expansions or declines^19,20^. Therefore, the use of vegetation proxies for past rainfall reconstructions is suitable in Mediterranean regions. In this study we aim to reconstruct the quantitative annual, winter and summer precipitation conditions from the southern Iberian Peninsula for the last 200 kyr using the fossil pollen data from Padul.

Quantitative paleoclimate reconstructions have previously been made using long sediment record data from different parts of the world^21–23^. However, one typical but often overlooked fact with the quantitative climate reconstructions is that results can be noisy due to the inherently high proportion of random variability in the data. It is therefore a challenge to separate true signal from noise in the records. The progress in statistical data analysis have provided solutions for this challenge, such as the use of the SiZer method in paleoclimate studies^24,25^. In this study, we use the newly developed SnSiZer method to identify not only the statistically significant changes in our quantitative precipitation reconstruction but also the relative magnitude of these oscillations.

We further compare our results with other records from the West/Central Mediterranean and West African region to discuss the similarities/differences between the identified paleoclimate periods from the penultimate glacial period to the Holocene and suggest the atmospheric-oceanic circulation mechanisms that may have caused the different humidity conditions.

### Regional setting, data and brief methodology

**Figure 1:**
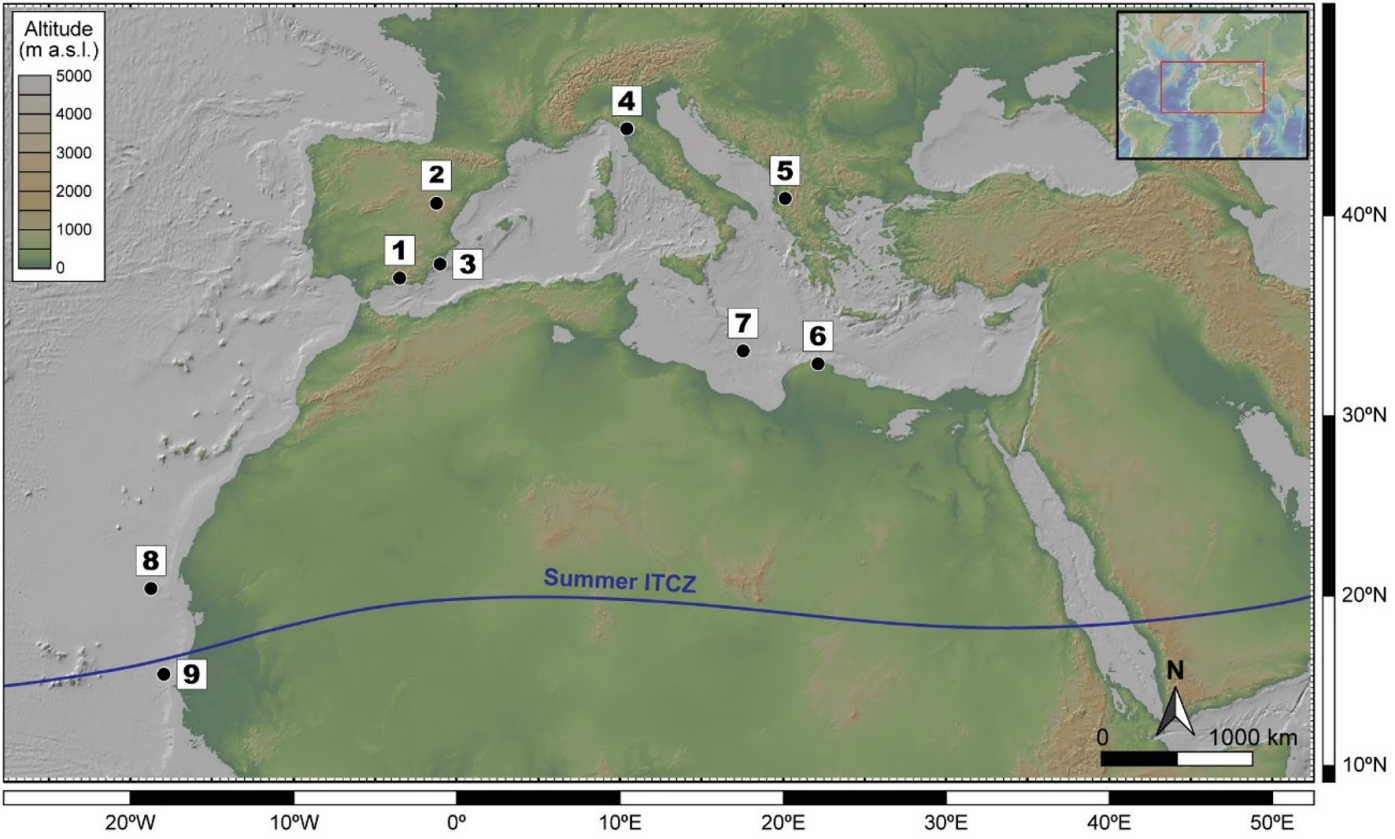
Map showing the paleoclimate records mentioned in this study: (1) Padul (Spain), (2) Villarquemado (Spain)^29,30^, (3) Victoria cave (Spain)^45^, (4) Corchia cave (Italy)^41–44^, (5) Lake Ohrid (Macedonia/Albania)^11^, (6) Susah cave (Libya)^2,46^, (7) 64PE349-8 record (offshore Libya)^47^, (8) GeoB7920-2 record (offshore Mauritania)^58^ and (9) GeoB9508-5 record (offshore Senegal)^51^. The present-day position of the summer ITCZ is shown. Map obtained from GeoMapApp (www.geomapapp.org)^95^/CC BY.

The Padul wetland is located in the extensional Padul-Nigüelas basin in the western margin of the Sierra Nevada range, in southern Iberian Peninsula (37°00′39″N, 3°36′14″W, 726 m a.s.l.) (Fig. 1). The 42.64-m-long and continuous Padul-15-05 sediment core was retrieved from the site in 2015 and spans the last ca. 200 kyr^26,27^. This region is characterized by a semiarid Mediterranean climate with summer drought and strong continentality. The present mean annual precipitation and temperature recorded between 2001 and 2022 at the agroclimatic meteorological station from Padul is 426 mm/yr and 15.5 °C, respectively (www.juntadeandalucia.es/agriculturaypesca/ifapa/riaweb).

**Figure 2:**
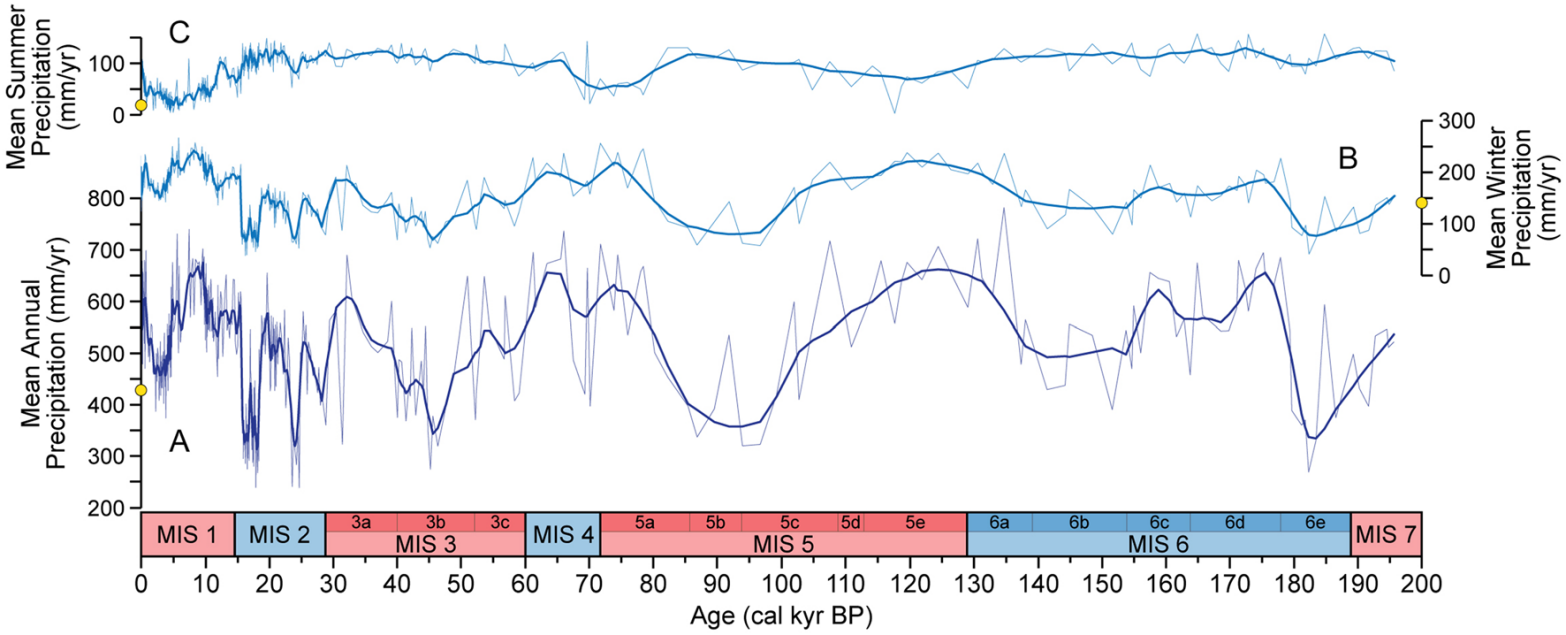
Quantitative reconstruction results from the Padul record for the last 200 kyr: (**A**) Mean annual precipitation (MAP), (**B**) mean winter precipitation (MWP) and (**C**) mean summer precipitation (MSP) reconstructions (in mm/yr). Light lines show the raw data, whereas the darker lines indicate the locally estimated scatterplot smoothing (LOESS) (span 0.02). Yellow dots show the present mean annual, winter and summer precipitation recorded at the agroclimatic meteorological station from Padul between 2001 and 2022 (426, 141 and 12 mm/yr, respectively).

In order to identify the relevant humid periods of the southern Iberian Peninsula and western Mediterranean, we generated quantitative reconstructions of the mean annual precipitation (MAP), mean winter precipitation (MWP, December-January–February) and mean summer precipitation (MSP, June–July–August) (Fig. 2) based on the combined Padul fossil pollen data from previous studies^18,26,28,68^. In addition, for comparison we also performed the MAP reconstruction from the Villarquemado pollen record (NE Iberia)^29,30^ following the same methodology as in Padul, and thus reducing the bias produced by using different training-sets from different studies. For detailed methodology, see “Methods” section.

## Results

### South Iberian and West Mediterranean precipitation changes

**Figure 3:**
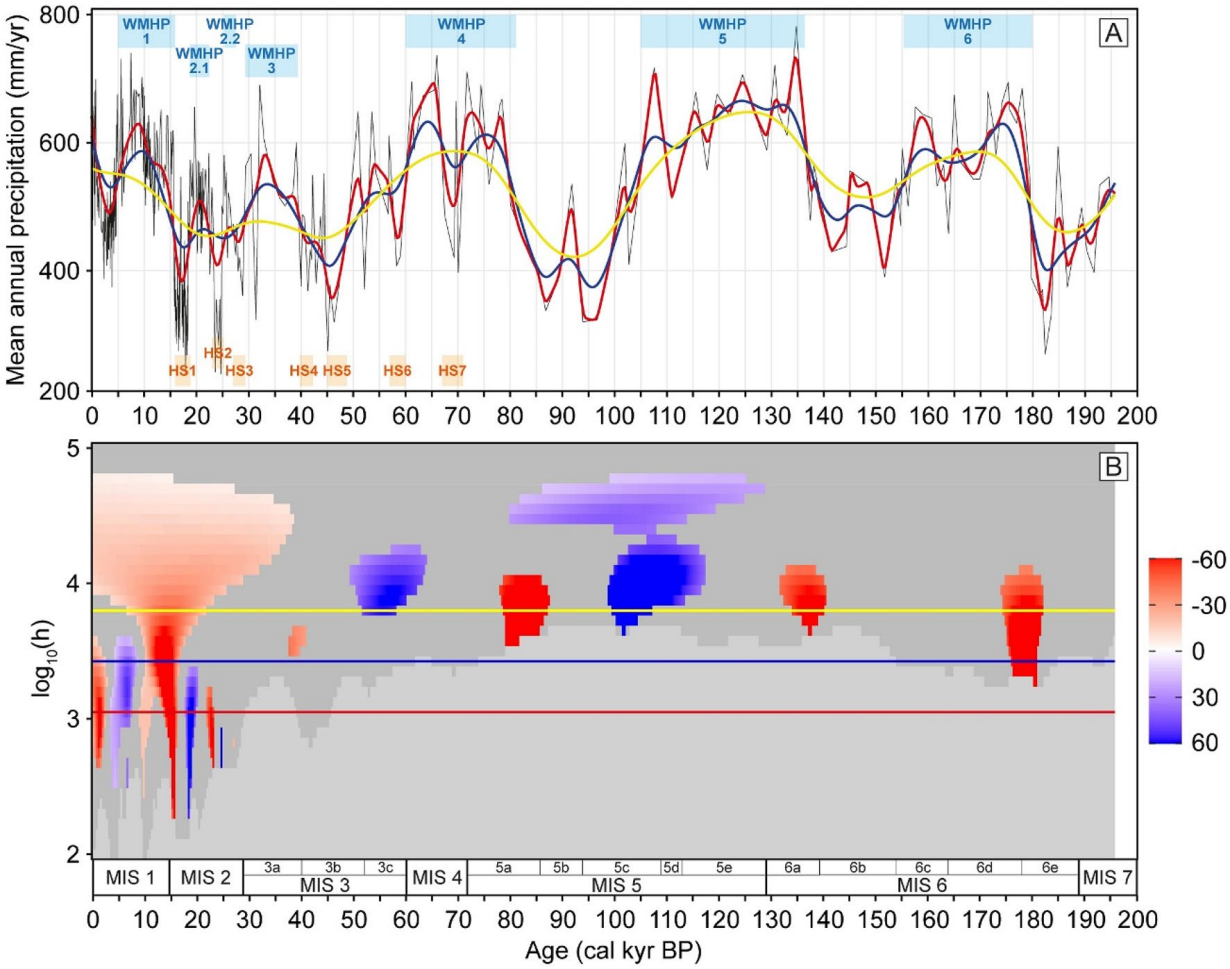
SnSiZer analysis developed on the Padul MAP reconstruction, showing the statistically significant changes of the data for the last 200 kyr. On the top panel (**A**), we show the raw MAP reconstruction with different Nadaraya-Watson smoothing levels (red-blue-yellow lines) along with the WMHPs and HSs. On the bottom panel (**B**), reading from past to present (from right to left), the red and blue colors indicate the statistically significant precipitation increases and decreases, respectively. The vertical axis represents the level of smoothing in logarithmic units and the horizontal red-blue-yellow lines the smoothing levels as in the upper panel. Dark grey areas represent no significant changes and light grey areas represent where the sampling resolution is too low.

**Figure 4:**
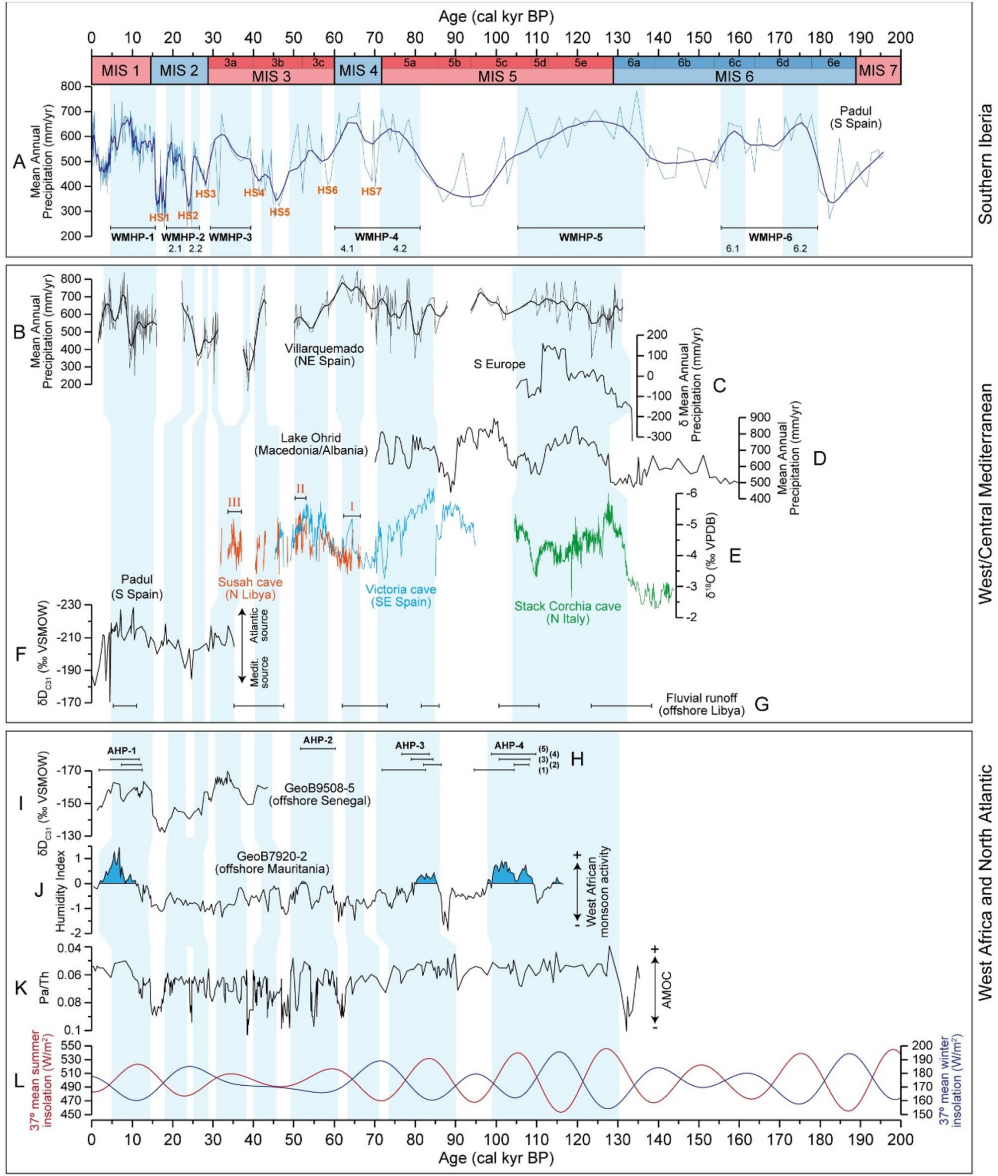
Comparison of the Padul MAP reconstruction with other multiproxy records for the last 200 kyr BP. Top panel (southern Iberia): (**A**) Reconstructed Padul MAP (mm/yr) along with the WMHPs (black horizontal lines) and HSs. Middle panel (other West/Central Mediterranean records): (**B**) our Villarquemado MAP reconstruction (mm/yr) (NE Spain), (**C**) Average MAP change (mm/yr) for southern Europe^40^, (**D**) MAP reconstruction from Lake Ohrid (Macedonia/Albania)^11^, (**E**) δ^18^O from Susah cave (N Libya)^2,46^, Victoria cave (SE Spain)^45^ and Corchia cave (N Italy)^41–44^ (note inverted scale), (**F**) δD_C31_ from Padul^31^ (note inverted scale), (**G**) fluvial runoff pulses from the 64PE349-8 record (offshore Libya)^47^. Bottom panel (West Africa, North Atlantic and insolation): (**H**) age-ranges of African Humid Periods (black horizontal lines) identified by different studies: (1) Ehrmann et al.^96^, (2) Grant et al.^97^, (3) Skonieczny et al.^98^, (4) Ziegler et al.^99^ and Pausata et al.^48^, and (5) Kinsley et al.^100^, (**I**) δD_C31_ from the GeoB9508-5 record (continental slope off Senegal)^51^ (note inverted scale), (**J**) Humidity index from the GeoB7920-2 record (offshore Mauritania)^58^, (**K**) Pa/Th from the North Atlantic CDH19 record^61^ (note inverted scale), (**L**) 37°N mean summer and winter insolation.

The Padul MAP, MWP and MSP reconstructions show the annual and seasonal precipitation conditions over the last 200 kyr, with a number of wet and arid periods. The MAP values oscillate between ca. 200 and 800 mm/yr, whereas the MWP and MSP vary between ca. 50–250 and 0–150 mm/yr, respectively (Fig. 2). In the novel SnSiZer analysis of the MAP, reading the graph from past to present, the red color indicates the statistically significant increases and blue color statistically significant decreases in precipitation, with the shading of the coloring indicating the strength of the change (Fig. 3). The frequency of the statistically significant changes is higher for the last 30 kyr due to the higher sample resolution in the original fossil pollen data. The statistically significant precipitation increases are termed West Mediterranean Humid Periods (WMHPs), similar to that observed by García-Alix et al.^31^ for the Holocene humid period recorded in the West Mediterranean between 15.5 and 5 kyr BP. Therefore, we obtained six main WMPHs, three of them divided into 2 sub-phases: WMHP-6 (180–155 kyr BP; WMHP-6.2 at 180–171 kyr BP and WMHP-6.1 at 161–155 kyr BP), WMHP-5 (136–105 kyr BP), WMHP-4 (81–60 kyr BP; WMHP-4.2 at 81–71 kyr BP and WMHP-4.1 at 66–60 kyr BP), WMHP-3 (39–29 kyr BP), WMHP-2 (27–18.5 kyr BP; WMHP-2.2 at 27–25 kyr BP and WMHP-2.1 at 23–18.5 kyr) and WMHP-1 (15.5–5 kyr BP) (Figs. 3 and 4A).

The highest Padul MAP of the last 200 kyr (ca. 800 mm/yr) corresponds to the beginning of the last interglacial period in Padul at 135 kyr BP (subject to age uncertainties), whereas the lowest values (ca. 200 mm/yr) date to 183, 45, 25–23 and 18.5–15.5 kyr BP (Figs. 2A and 3). In addition, our precipitation reconstructions show seasonal differences in the precipitation conditions in southern Iberian Peninsula. MWP presents a similar trend to MAP, suggesting that the annual precipitation in this area was mainly controlled by winter precipitation (Fig. 2B). On the contrary, MSP shows an anticorrelation to MAP and MWP, with higher summer precipitation values during glacial periods (e.g., MIS 6, 3 and 2) and lower values during interglacials/interstadials (MIS 5e, 5a and 1). As we can observe in the EMPDv2 training-set (Figs. S1 and S2), this is a result of the higher abundance of *Pinus* and *Artemisia* (the main taxa during glacial periods in Padul) under higher summer precipitation conditions compared to the winter season (Fig. S2). In particular, the present-day *Pinus* species growing at high altitudes in the Sierra Nevada range (i.e., *Pinus sylvestris* and *P. nigra*) also present the same seasonal trend (Fig. S2). Moreover, the present-day steppe ecosystems from high-altitudes in the northwestern Africa with the presence of *Artemisia herba-alba* as the main taxon, which is also an important component of steppe flora in the Iberian Peninsula^32^, are characterized by a west Mediterranean rainfall sub-regime with significant amount of summer precipitation^33^. According to this, the slightly higher glacial MSP values compared to interglacial/interstadials could be explained by the presence of both alpine *Pinus* species and *Artemisia* at low-altitude areas from Sierra Nevada (e.g., Padul) due the downward movement of the high-altitude vegetation belts during glacial periods^26^. However, since some *Pinus* species (e.g., *P. sylvestris*) currently occur over wide areas and under different climate zones, the summer precipitation results could be an artefact for the Mediterranean climate reconstruction and should be taken with caution. Also, the human activity has affected the vegetation in the Padul region during the last ca. 1500 years^18^, and therefore, the precipitation reconstruction for this period may be masked and not fully reliable.

In addition to the humid periods observed in southern Iberia, the Padul precipitation reconstruction is also characterized by repeated drought events, which last mostly 2–5 kyr (Figs. 3 and 4A). The pollen composition during these events is characterized by declines in the Mediterranean forest and peaks of *Artemisia*, Amaranthaceae, Asteraceae and *Ephedra* (Fig. S3). These are all typical components of the desert and steppe ecosystems, with highest pollen percentages in the modern pollen samples from northern Africa in our calibration model. These events generally match the timing of arid Heinrich Stadials (HSs)^34,35^ (Fig. 4A) and some of them are statistically significant under the SnSiZer analysis, such as the drought events at 60–57 (HS6), 25–23 (HS2) and 18.5–15.5 kyr BP (HS1) (Fig. 3).

## Discussion

### Comparison of the WMHPs with other West/Central Mediterranean records

Similar to Padul, which is the oldest southernmost continental archive in the Iberian Peninsula (spanning the last ca. 200 kyr), Villarquemado is the unique northeastern Iberian lacustrine record covering the last 135 kyr. This fact makes their comparison suitable, and essential, to explore paleoclimatological patterns.

Thus, when the Padul and Villarquemado MAP reconstructions are compared, the Villarquemado MAP reconstruction shows the highest MAP during the WMHP-4.1 and the Early Holocene, with values ranging between 700 and 800 mm/yr (Fig. 4B). The WMHP-5 and WMHP-4.2 in Villarquemado do not show such high precipitation conditions as in Padul. This may be caused by the extremely high continental climate affecting Villarquemado during interglacial periods and high-seasonality phases^12^, which produces different climate conditions, different local and regional paleoenvironment and edaphic development, different vegetation dynamics and, consequently, a different pollen composition from that of Padul. Both Padul and Villarquemado MAP reconstructions match relatively well from MIS 5a to MIS 3c (ca. 85–50 kyr BP). However, during the MIS 3a, the increasing precipitation of the WMHP-3 in Padul does not match with the pollen sterile phase in Villarquemado at 37–31 kyr BP. The poor pollen preservation scenario in Villarquemado was interpreted as consequence of oxidation processes and arid conditions suggested by sedimentological and geochemical data^12^. This could be related with the different biogeographical features and the different sedimentation conditions in both sites during this period, which affected the pollen and geochemical compositions.

Only few pollen records cover periods older than the Eemian in the Mediterranean region^30,36–39^. Sediment lake records older than 100 kyr exist only in the northern side of the Mediterranean and the only available quantitative precipitation reconstructions come from Lake Ohrid (Macedonia/Albania)^11^ and Lago Grande di Monticchio (including an stack for southern Europe)^40^, which span barely from 160–70 and 135–105 kyr BP, respectively. The MAP reconstruction from Lake Ohrid shows high precipitation values at 128–112 and 85–70 kyr BP, corresponding with the mid WMHP-5 and WMHP-4.2 at Padul (Fig. 4A,D). The average mean annual precipitation change based on four south European records (including Lago Grande di Monticchio)^40^ presents a gradually increasing precipitation trend from 135 to 110 kyr BP, with the highest values between 117 and 111 kyr BP (Fig. 4C), suggesting similar high precipitation conditions to that reconstructed for Padul during the WMHP-5. However, increasing precipitation in Lake Ohrid between 103 and 90 kyr BP does not match with the precipitation trends in Padul, suggesting different climate factors affecting the central and western Mediterranean, or age uncertainties between records during this period.

Speleothem records also provide information about moisture changes from the study region and can be compared with the Padul MAP reconstruction and the WMHPs. The stacked δ^18^O from Corchia cave in northern Italy covering between 145 and 105 kyr BP^41–44^ shows decreasing isotope values from the end of the penultimate glacial to the last interglacial period, suggesting increasing precipitation conditions as observed in Padul from the MIS 6 to WMHP-5 (Fig. 4A,E). With respect to the Victoria cave in southeastern Spain^45^, the decreasing isotope values at 95–87 kyr BP related with a humid phase is not observed in Padul (as previously discussed with Lake Ohrid). During the period from 85 to 45 kyr BP the speleothem isotope record matches with the Padul reconstruction, and thus with the WMHP-4.2 and 4.1, and the following not-statistically significant (under SnSizer) wet-phase at 57–49 kyr BP (Fig. 4A,E).

The paleohydrological record from the Susah cave in northern Libya is particularly useful for identifying precipitation patterns in the southern part of the Mediterranean and northern Africa, as the growth of speleothems under desert conditions is highly dependent on the surplus of effective precipitation and thus humid conditions in northern Africa^2,46^. The main phases of sustained speleothem growth (I, II and III in Fig. 4E) date to 65–61, 52.5–50.5 and 37.5–33 kyr BP, corresponding with the timing of the WMHP-4.1 (66–60 kyr BP), the wet-phase at 57–49 kyr BP and the early WMHP-3 (39–29 kyr BP), respectively (Fig. 4A,E). In addition, in a 160 kyr marine sediment core offshore Libya the sediment provenance indicators show a number of periods when the fluvial network from the northern Sahara was activated, suggesting periods of humid climate in the southern Mediterranean region^47^. These humid periods date to 138–123 (corresponding to early WMHP-5), 110–100 (corresponding to late WMHP-5), 86–81 (corresponding to early WMHP-4.2, subjected to age uncertainties), 73–62 (corresponding to WMHP-4.1, including HS7), 48–35 (corresponding to early WMHP-3 and wet-phase at 45–42 kyr BP, including HS4) and 11–5 kyr BP (corresponding to WMHP-1) (Fig. 4A,G). We therefore conclude that our WMHPs identified in Padul correlate relatively well with other pollen, isotope and paleohydrological results, and thus with the humid periods observed in western and northern Mediterranean as well as with the southern Mediterranean (northern African) areas.

### WMHPs and AHPs: relationship and paleoclimatic interpretation

Reconstructions of high humidity during the so-called Green Sahara periods are well documented in the studies from Sahara and the African west coast and associated with stronger West African Monsoon (WAM)^1^, which regulates the annual rainfall amount and the climatological rainfall patterns. The humid conditions during the AHPs are mostly caused by periods of enhanced WAM and associated transport of moist air masses (mainly during summer times) from the equatorial Atlantic towards the northern parts of the Sahara^48^, whereas northern Africa is also affected by the southward shift of the Mediterranean winter precipitation system^3^. The most recent Holocene AHP (ca. 11–5 kyr BP) has been intensively investigated^49,50^, but very few studies show the relationship between the humid periods in Sahara and the Mediterranean region for older periods^3^.

At the orbital-scale and during high-seasonality (high summer and low winter insolation), the WMHPs in southern Iberia are in-phase with the enhanced WAM activity, as observed with the increasing Humidity Index in West Africa at 110–100 kyr BP (AHP-4 corresponding to late WMHP-5), 85–80 kyr BP (AHP-3 corresponding to WMHP-4.2), 60–50 kyr BP (AHP-2 corresponding to the wet-phase at 57–49 kyr BP) and 11–5 kyr BP (AHP-1 corresponding to WMHP-1) (Fig. 4A,H,J). Similarly, the hydrogen isotopic composition of terrestrial leaf waxes (δD_C31_) from the continental slope off Senegal for the last 45 kyr BP^51^ show parallel trends with respect to the MAP and the δD_C31_ records from Padul^31^ (Fig. 4A,F,I), suggesting that climate in West Africa and West Mediterranean seems to be controlled by the same orbital factors. These parallel responses of the humid periods in the West Mediterranean and West Africa during high summer insolation could be related with the strong Atlantic Meridional Overturning Circulation (AMOC) activity, generating a southward displacement of the Atlantic storm tracks (westerlies) and providing high moisture conditions over the western Mediterranean and the Iberian Peninsula, especially during winter-time^52,53^. Simultaneously, the increasing summer insolation intensified the land-sea temperature gradient and the summer monsoonal circulation in Africa, affecting the northward shift of the Intertropical Convergence Zone (ITCZ), a northward extension of the rainfall and the greening of Sahara^54,55^. However, proxy-based studies and climate model simulations show that the WAM was enhanced and the precipitation increased in the southwestern part of the Sahara, whereas the northwestern Sahara remained mostly dry. This is supported by stable isotope evidence from speleothems in Morocco, showing increased precipitation in the southern part but not in the northern part of the country^56,57^.

The Padul MAP and MWP reconstructions show that the high precipitation conditions in southern Iberian Peninsula occurring during interglacials and interstadials, such as the last interglacial, MIS 5a and Holocene (WMHP-5, 4.2 and 1), were mainly associated to winter precipitation, whereas the summer precipitation was low (Fig. 2). This indicates that the main water-source in western Mediterranean during these periods of high summer insolation was controlled by the Atlantic wintertime storm track activity. This is supported by the hydrogen isotopic composition of terrestrial leaf waxes of the last 35 kyr, showing that although the correlation between the δD_C31_ from Padul and West Africa is high, the differences in absolute values point to different precipitation sources^31^ (Fig. 4F,I).

In contrast, under low-seasonality, the moderate precipitation periods observed in Padul and in other West/Central Mediterranean records, such as the WMHP-4.1 (growth-phase I in Susah cave, more negative isotope values in Victoria cave, high MAP in Villarquemado), WMHP-3 (growth-phase III in Susah cave) and WMHP-2 (moderate MAP in Villarquemado during the early WMHP-2), correlate with low rainfall or even drought conditions in the humidity records from western and eastern Africa^58,59^ (Figs. 4A,B,E,J and S7). During these low-seasonality phases, moderate-to-high precipitation conditions still affected the Iberian Peninsula and other Mediterranean areas, whereas the West African records suggest low monsoon activity. It is well-known that the high summer insolation affected the increasing precipitation conditions in the Mediterranean region^60^. However, even if the summer insolation was low, the AMOC remained active, as shown by the relatively low Pa/Th values from the North Atlantic^61^ (Fig. 4K,L). This could have provided moderate Atlantic winter moisture conditions to the Iberian Peninsula and the Mediterranean area during these low-seasonality periods, whereas the same low summer insolation resulted in dry Sahara periods, as insolation acted as the main precipitation control for the summer WAM activity and global monsoon systems^54^.

The seasonal distribution of rainfall in the past may not follow the present seasonal precipitation patterns in the region, hence the effect of the local Mediterranean rainfall flux during low summer insolation and glacial periods could have played an important role in the western Mediterranean precipitation. The winter precipitation is the main factor controlling the forest expansion in this area^19,20^, and therefore, the reduction of the tree cover and steppe expansion do not necessarily imply a reduction in the summer precipitation^62^. Our high MSP reconstructed values of the glacial periods (e.g., MIS 6, 3 and 2) (Fig. 2) are strongly influenced by the increasing *Pinus* percentages in the Padul fossil record during these periods and the high abundance of *Pinus* in the training-set during high recent summer precipitation conditions (see “Results” and Fig. S2). The lower temperature and evapotranspiration conditions of the glacial periods could have favored the pine forest expansion, and at the same time, providing higher MSP values compared to interglacials/interstadials. The relative higher glacial MSP could also suggest the different contribution of the summer moisture compared to present conditions, similar to that observed and interpreted in other Mediterranean records during the last deglaciation and the Holocene^62,63^. In addition, periods of North Atlantic cooling in the western Mediterranean during the last 12 kyr resulted in winter rainfall minima due to the northward shift of the humid westerlies^64^. Similar weakening of the westerlies could have also happened during glacial periods the Iberian Peninsula, providing the perfect conditions for higher MSP related with higher contribution of the local Mediterranean summer rainfall-source. This is supported by the hydrogen isotopic composition of leaf waxes in the Padul record during the last 35 kyr, suggesting a higher influence of the local Mediterranean rainfall with respect to the Atlantic source during the glacial period^31^.

Studies on oxygen isotopes in speleothem records from the Mediterranean region can also inform about the amount of precipitation and the moisture-source of the rainfall (Mediterranean *vs* Atlantic)^65^. The influence of the air mass source has an effect on the δ^18^O record from recent Iberian speleothems, showing more negative values during enhanced Atlantic fronts and less negative values during periods with higher influence of local Mediterranean rainfall^66,67^. This could also explain the local Mediterranean rainfall influence on the high Padul MAP conditions during the WMHP-4.1, presenting less depleted δ^18^O values in Susah (growth-phase I) and Victoria caves with respect to the following humid periods under higher summer insolation, such as the wet-phase at 57–49 kyr BP (growth-phase II in Susah) and WMHP-3 (growth-phase III in Susah) (Fig. 4A,E). Therefore, the local Mediterranean convective precipitation could have also played an important role during specific periods within the last glacial cycle, especially during the low summer insolation phases.

## Conclusions

The quantitative precipitation reconstruction from the fossil pollen data from Padul provides the mean annual, winter and summer precipitation changes from southern Iberian Peninsula for the last ca. 200 kyr, being the longest continuous continental quantitative paleoclimate reconstruction from the western Mediterranean region. The newly developed SnSiZer method was applied to identify the statistically significant precipitation changes as well as the relative magnitude of the humidity oscillations. Using this approach, we identify six main WMHPs: WMHP-6 (180–155 kyr BP), WMHP-5 (136–105 kyr BP), WMHP-4 (81–60 kyr BP), WMHP-3 (39–29 kyr BP), WMHP-2 (27–18.5 kyr BP) and WMHP-1 (15.5–5 kyr BP).

Based on our precipitation reconstruction, with the identified WMHPs and their comparison with West/Central Mediterranean and West African records, we conclude that during high seasonality, both West/Central Mediterranean and West African climates are in phase, with roughly coeval humid periods in both regions. In contrast, west Mediterranean and west African records are out of phase during low seasonality. This could be explained by the westerlies and the influence of the local Mediterranean rainfall flux affecting the Mediterranean region, providing moderate precipitation conditions (e.g., WMHP-4.1, WMHP-3), whereas West Africa was characterized by drier and even drought conditions.

## Methods

### Chronological control

The chronological control of the Padul-15-05 record is based on forty-two accelerator mass spectrometry (AMS) radiocarbon dates (including three specific compound radiocarbon dates), four amino acid racemization (AAR) dates (three of the dates at 133, 116 and 107 kyr BP averaged to 118 kyr BP) and two different sediment accumulation rates (SAR, for both peat and carbonate/marl lithologies extrapolated from the top of the core), resulting in a sediment and climate record of the last 197 cal kyr BP^26,27^.

### Fossil pollen record

We focus on the last ca. 200 kyr from the Padul pollen record, containing a total of 438 fossil pollen samples. The mean data resolution of the Padul pollen data, and therefore, of the precipitation reconstructions for the last 200 kyr is 447 yrs, being higher for the last 30 kyr (96-yr resolution). The Padul pollen sequence was previously discussed qualitatively by Ramos-Román et al.^18,68^ and Camuera et al.^26,28^. The simplified pollen diagrams with the relative abundances of the most important taxa from Padul are shown in the supplementary Fig. S3. The tree/shrub sequence in Padul is mainly dominated by *Quercus* (evergreen and deciduous), *Pinus*, Cupressaceae, *Pistacia* and *Olea*, whereas herbs and grasses are primarily composed by Poaceae, *Artemisia*, Amaranthaceae, Asteraceae Cichorioideae, Asteraceae Asteroideae and Ericaceae.

### Modern pollen dataset

The modern pollen dataset used for quantitative paleoclimate reconstructions is based on the new Eurasian Modern Pollen Database (EMPDv2)^69^, resulting in a total of 8174 modern pollen samples (Fig. S1). The pollen taxonomy has been harmonized based on the EMPDv2 and the minor taxa/species with low relative abundances were integrated within major taxa/genera/families with the help of the Plants of the World online database (http://www.plantsoftheworldonline.org) and the Integrated Taxonomic Information System (http://www.itis.gov). Aquatic (e.g., *Myriophyllum*, *Lemna*, *Nuphar*) and cultivated plants (e.g., *Avena*, *Zea mays*) were removed, assuming that the distribution of these plants could be affected by other factors not related to climate. With respect to the present climate parameters, the mean annual-winter-summer precipitation was obtained from the WorldClim v2.1 database under a 30 s resolution (www.worldclim.org)^70^. For the statistical performance of the training-set and reconstructions, see “Testing reconstruction reliability” section.

### Numerical analysis of the climatic variables and modern pollen data

Constrained ordination methods were applied to quantify the relevance of the precipitation variables (annual, winter and summer) that account for the distribution of modern pollen assemblages (Fig. S4). The canonical correspondence analysis (CCA) based on a unimodal method and the redundancy analysis (RDA) based on a linear method were carried out with the permutation test involving 999 permutations. In order to reduce the large effect of species/variables with many zero values, the CCA was developed under the option “downweigh rare species”. The data showed a gradient of 4.6 standard deviation units, making the CCA unimodal method more appropriate^71^. However, to provide a wider range for the proportion of variance explained by the climate variables, we have included the results of both CCA and RDA. The analyses show that the proportion of variation in the pollen data explained by the precipitation variables ranges between 7.09 and 11.59%. The CCA and RDA were implemented using the Canoco 5.12 software^72^.

In addition, the variance inflation factor (VIF) was run in order to measure the collinearity of each variable with the other two^73^. VIF values over 20 have been used as threshold for high collinearity in pollen-climate studies^74,75^. The VIF values for our MAP, MWP and MSP are 38.59, 22.95 and 9.37, respectively, indicating that annual and winter precipitations are highly collinear climate variables in our dataset. The high collinearity, as explained in “Results”, is a consequence of the high influence of the winter precipitation in the total amount of annual precipitation in the region, as well as the main control for the forest expansion or declines^19,20^. Despite the high collinearity between MAP and MWP and although the main goal of the paper focuses on the MAP, in order to observe the quantitative seasonal precipitation values, we have also included the MWP and MSP reconstructions in Fig. 2. The VIF analysis was carried out using the R software^76^ under the Vegan package^77^.

### Quantitative climate reconstruction method

Multivariate calibration methods are commonly used for quantitative paleoclimate reconstructions, including the Weighted Averaging method (WA)^78,79^, Partial Least Squares method (PLS)^80^ and Weighted Averaging-Partial Least Squares method (WA-PLS)^81,82^. In this study, the transfer function method under the WA-PLS regression technique was used on a total of 389 harmonized pollen taxa to derive quantitative climate reconstructions. The non-linear WA-PLS method assumes that each taxon has a unimodal distribution with respect to climate parameters and it is relatively robust to spatial autocorrelation^83^.

The pollen-based transfer function from Padul was developed using the C2 software under the version 1.7.7^84^. In order to reduce the noise of the data, square-root species transformation was used on the pollen training-set. For the construction of WA-PLS regressions, a total of 5 components were run, but we used the two-component WA-PLS model under the leave-one-out cross-validation method. Increasing the number of components produces a decrease in the root mean squared error, but can result in overfitting the data, and therefore, a decrease of the model predictive value^81^.

### Testing reconstruction reliability

The reliability of the quantitative reconstructions of Padul was assessed using the performance statistics of the WA-PLS-based transfer function model under cross-validation, including the coefficient of determination (*R*^2^), the root mean square error of prediction (RMSEP) and the maximum bias. The two-component WA-PLS of the MAP, MWP and MSP provided *R*^2^ values of 0.51, 0.47 and 0.65, respectively. For the MAP, MWP and MSP reconstructions, the RMSEP show values of 284, 102 and 62 mm/yr, whereas the maximum bias present values of 2318, 767 and 278 mm/yr, respectively (Fig. S5).

We also assessed the goodness-of-fit analysis in order to test the similarity between fossil and modern pollen samples, showing us which samples have good similarities with modern samples from the training-set^85^. In particular, this analysis is based on the pair-wise distribution of squared-chord distances^86^ between the Padul fossil pollen samples and best analogues in the modern training-set. A “good-analogue” is considered when the minimum dissimilarity coefficient (squared-chord distance) between each fossil sample and samples from the training-set is lower than the 5th percentile of all distances, whereas distances between the 5th–10th percentile and larger than the 10th percentile are regarded as “fair-analogue” and “non-analogue” assemblages, respectively^85^. The analogue evaluation suggests a good match between the fossil pollen samples from Padul and modern pollen samples. The analysis shows that the good analogues represent 78.77%, the fair analogues 19.86% and the non-analogues 1.37% (Fig. S6).

### SnSiZer for detection of significant features in time series

The significance of the trends and anomalies in the mean annual precipitation reconstruction from Padul was statistically analyzed using a SnSiZer, a new version of the original SiZer analysis^24^, which is an inference tool that has shown its usefulness for example in ecology^87,88^. SnSiZer was developed under the R Studio version 1.2.5019 (www.rstudio.com) by modifying the source-code of the sizer package^89^.

When used for time series, the original SiZer and the novel SnSiZer analysis apply a nonparametric smoothing to a signal and detect the time intervals with significantly increasing or decreasing smooth. A wide range of smoothing levels are used for revealing the salient features in the signal at all frequencies. Thus, in the SnSizer graph, when the smooth, at a given smoothing level (red, blue or yellow lines in Fig. 3B), cut the statistically significant increasing (red) or decreasing (blue) features, it means that those changes are statistically relevant under that level of smoothing.

In the conventional SiZer, a wide range of smoothing levels are used for revealing the salient features in the signal at all frequencies. When applied for time series, the results are visualized using a color graph where the time is on the horizontal axis and the smoothing level is on the vertical axis. The log_10_(h) has not unit and represents the smoothing level. Usually, the values for smoothing are large and the log_10_ of these values must be taken. For each pixel, red, blue and grey colors represent the significance of the derivative of the smooth for the corresponding time point and scale. For evaluating the strengths of the increases and declines, the intensity of colors could be based on the relative magnitude of the derivative of the smooth. However, smoothing dilutes derivatives, and therefore the strengths of the increases and decreases couldn’t be compared between the scales. For allowing a fair comparison of the relative magnitudes of derivatives between smoothing levels, it is possible to use the so-called scale-normalized derivatives, where the derivative of the smooth is scaled with respect to the smoothing level^90^. Such derivatives can be used in data analysis in various ways, for example for estimating characteristic feature sizes in time series^91^.

### Scale-normalized derivatives

Mathematically, the scale-normalized derivatives used for the SnSiZer can be described as follows:

Let $K$ be the standard Gaussian density function and ${h}^{2}$ the variance. Let us consider a continuous signal $u(t)$, $t\in {\mathbb{R}}$. Then the convolution smooth $L(t,{h}^{2})$ of $u(t)$ is defined as:

$$L\left(t,{h}^{2}\right) :=\int \begin{array}{c}\infty \\ -\infty \end{array}u\left(t-z\right)\frac{K\left(\frac{z}{h}\right)}{h}dz=\left(u*{K}_{h}\right)\left(t\right),$$

where $${K}_{h}(.)=1/hK(./h)$$.

Smoothing reduces rough small-scale features revealing averaged features over longer and longer windows of $t$. This means that $\frac{\partial L\left(t,{h}^{2}\right)}{\partial t}\to 0$ as $h\to \infty$, i.e., smoothing suppresses derivatives, making the relative magnitudes of the derivatives useless as a measure of salience of a feature, e.g., peak or valley, in a signal.

As a remedy, Lindeberg^90^ introduced a so-called $\gamma$-normalized derivatives ${h}^{\gamma } \frac{{\partial }^{m} L(t,{h}^{2})}{\partial {t}^{m}}$, where $0<\gamma \le 1$ is a so-called normalization parameter and $m$ is the degree of the derivative. Here, we focus only on the case where $\gamma =1$ and $m=1$ and refer to such normalized derivatives as scale-normalized derivatives. In such derivatives the change in a smooth $L(t,{h}^{2})$ is not measured with respect to $t$ but instead with respect to normalized (dimensionless) coordinates $\xi =t/h$^92^ and therefore:

$$\frac{\partial L(t,{h}^{2})}{\partial \xi } = h \frac{\partial L(t,{h}^{2})}{\partial t}.$$

Hence, the scale-normalized derivative measures the change in the signal with respect to a temporal horizon that depends linearly $h$. While the relative magnitude of the ordinary derivative of the smooth declines with scale, the scale-normalized derivative remains unaffected, given that the scale and temporal positions match^90^. This means that the scale-normalized derivative treats signals with different scales fairly, enabling the comparison of the strength of the features of a signal over different scale horizons. In a time series framework, Lindeberg^90^ demonstrates further that with sinusoidal waves, the scale-normalized derivative depends only on the amplitude of the signal and not on its frequency.

The scale normalized derivatives of a Gaussian smooth correspond to taking convolution with ${L}_{1}$ normalized Gaussian derivative kernel, i.e. making continuous wavelet transformations of the signal with Gaussian first order derivative wavelet^90,93^.

When defining the scale-normalized derivatives based on observed time series, the Gaussian convolution smoother must be replaced with a discrete alternative, such as local linear regression or Nadaraya-Watson estimator (see Wand and Jones^94^). To ensure that the scale-normalized derivative remains bounded as the scale increases, the smoothing method must be chosen so that the smooth tends to constant as the scale increases. Hence, the Nadaraya-Watson smoother, that tends to the mean of the signal, is used here.

## Supplementary Information

Supplementary Figures.

## Data Availability

The mean annual, winter and summer precipitation results from Padul are freely available at the Pangaea data repository (https://doi.org/10.1594/PANGAEA.940006).
