# Supplementary material for: Past 200 kyr hydroclimate variability in the western Mediterranean and its connection to the African Humid Periods
Source: Sci Rep. 2022 May 31;12:9050. doi: 10.1038/s41598-022-12047-1 (PMC9156737; doi:10.1038/s41598-022-12047-1)
Supplement: Supplementary file 1 — Supplementary Figures. [file 41598_2022_12047_MOESM1_ESM.docx]

**SUPPLEMENTARY INFORMATION**

**Past 200 kyr hydroclimate variability in the western Mediterranean and its connection to the African Humid Periods**

Jon Camuera^1,2^*, María J. Ramos-Román^1^, Gonzalo Jiménez-Moreno^3^, Antonio García-Alix^2,3^, Liisa Ilvonen^1,4^, Leena Ruha^5,6^, Graciela Gil-Romera^7,8^, Penélope González-Sampériz^7^, Heikki Seppä^1^

^1^ Department of Geography and Geosciences, Faculty of Science, University of Helsinki, Helsinki, Finland

^2^ Andalusian Earth Sciences Institute (IACT, CSIC-UGR), Armilla, Granada, Spain

^3^ Department of Stratigraphy and Paleontology, Faculty of Science, University of Granada, Granada, Spain

^4^ Research Centre for Ecological Change, Organismal and Evolutionary Biology Research Programme, Faculty of Biological and Environmental Sciences, University of Helsinki, Helsinki, Finland

^5^ Natural Resources Institute Finland, Oulu, Finland

^6^ Research Unit of Mathematical Sciences, University of Oulu, Oulu, Finland

^7^ Pyrenean Institute of Ecology (IPE-CSIC), Zaragoza, Spain

^8^ Department of Ecology, Faculty of Biology, Philipps-Marburg University, Marburg, Germany

*Corresponding author. Email: [jcamuera@gmail.com](mailto:jcamuera@gmail.com)

**The Supplementary Information includes:**

Supplementary Figures S1 to S7

Supplementary references

**SUPPLEMENTARY FIGURES**


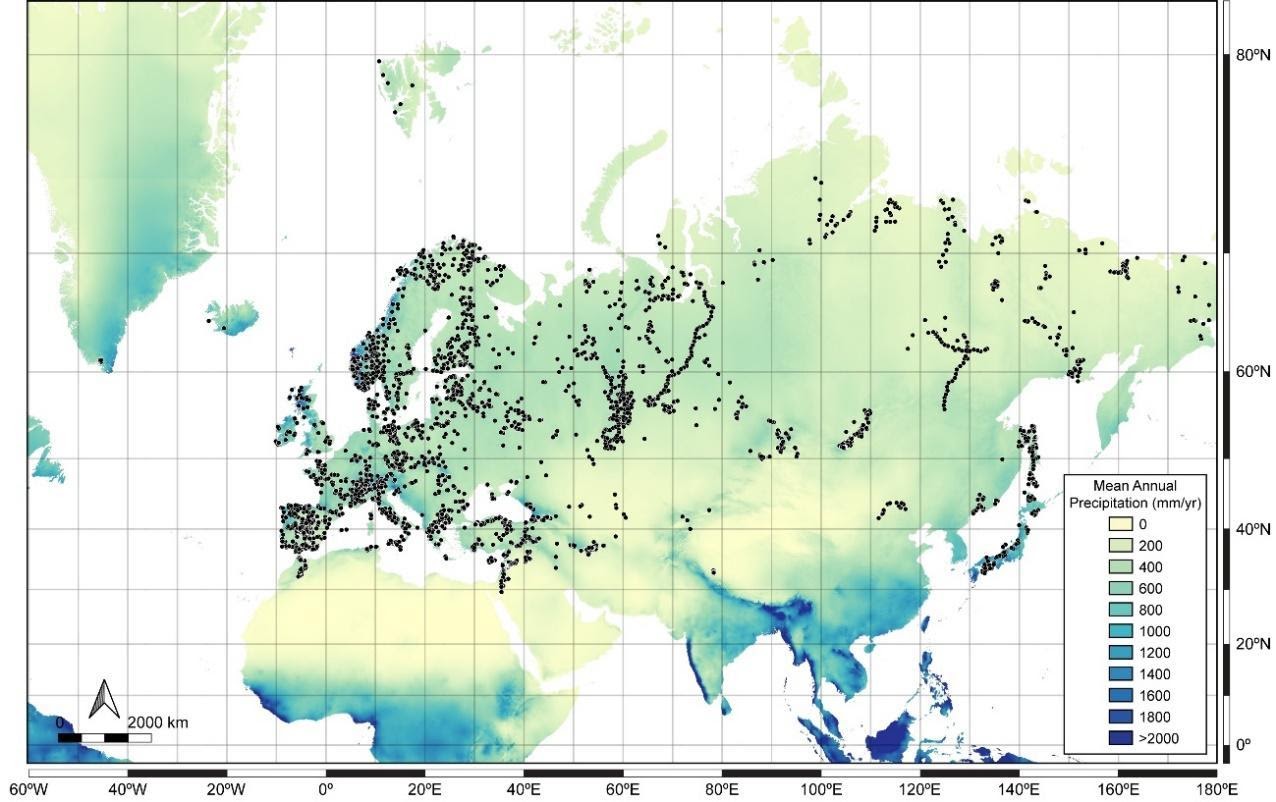


**Figure S1.** Map showing the raster layer of the mean annual precipitation (in mm/yr) (source WorldClim v2.1, average monthly precipitation for 1970-2000) along with the modern pollen sites (black dots) of the EMPDv2 (Eurasian Modern Pollen Database version 2)^1^.

**
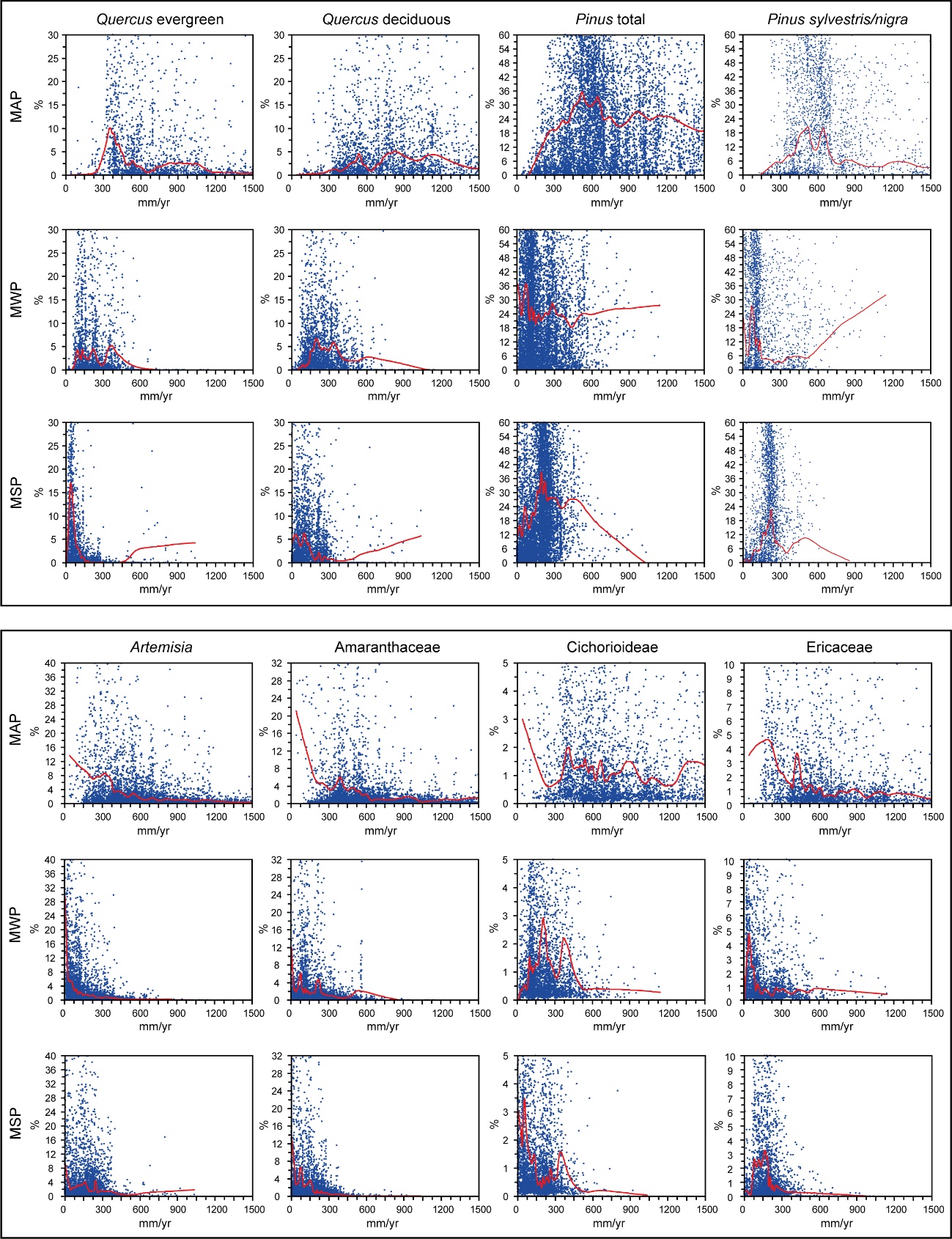
**

**Figure S2.** Abundances (in percentages) of the modern pollen data (top panel: trees; bottom panel: herbs) from the EMPDv2 with respect to the present mean annual precipitation (MAP), mean winter precipitation (MWP) and mean summer precipitation (MSP) values. These taxa were selected based on the main tree and herb taxa observed in the Padul fossil pollen record (Fig. S3). Blue dots represent sites in the EMPDv2. Red lines show the locally estimated scatterplot smoothing (LOESS) (span 0.01) of the percentages for each tree and herb taxon.


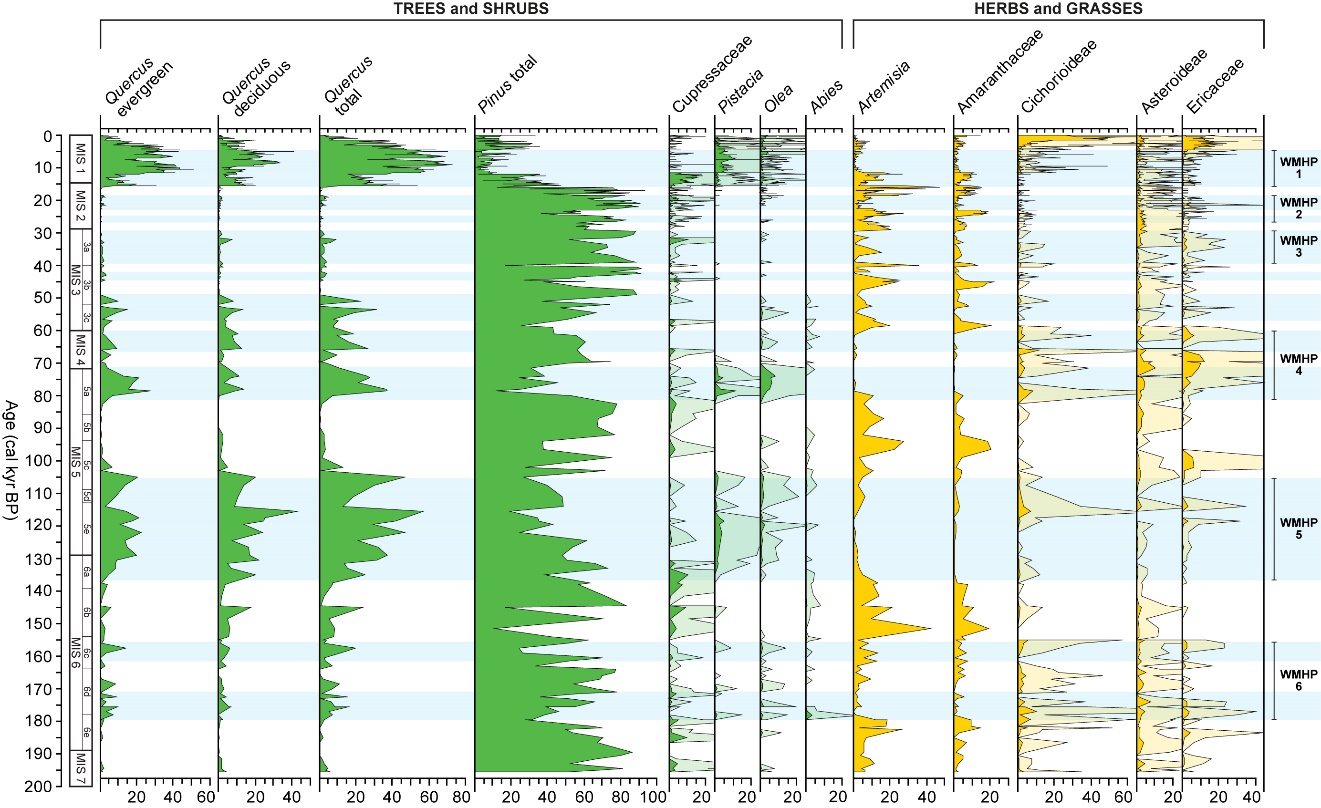


**Figure S3.** Schematic pollen diagram from Padul for the last ca. 200 kyr showing the percentages of the most characteristic pollen taxa, separated into trees/shrubs (green) and herbs/grasses (yellow). Percentages were calculated with respect to the total terrestrial pollen sum. Silhouettes in lighter color indicate 10x exaggeration percentages. For the original pollen study see Camuera et al. (2019)^2^. The blue shades show the humid periods as in Figure 4. On the right, the WMHPs are also shown.


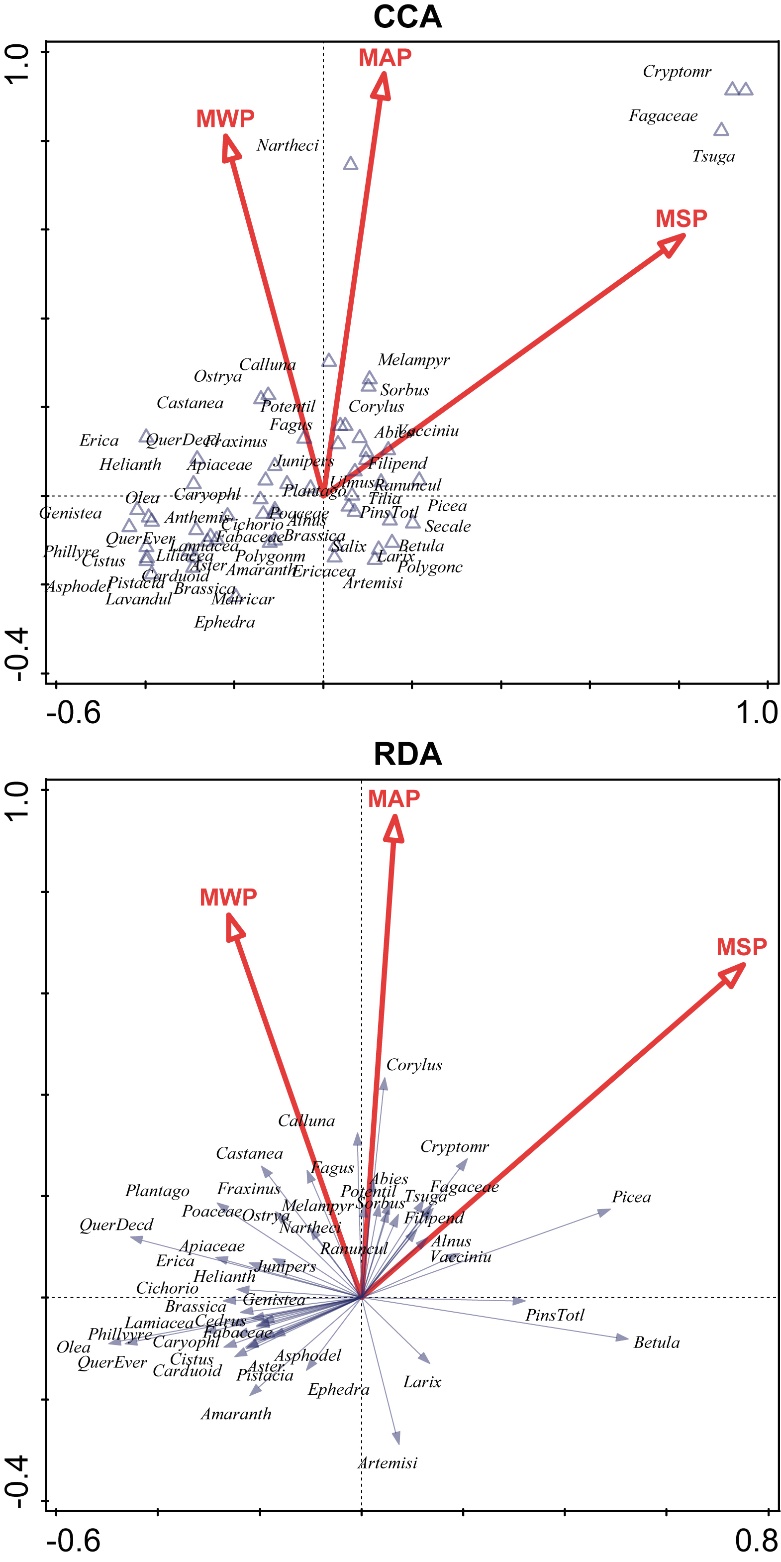


**Figure S4.** The canonical correspondence analysis (CCA) and redundancy analysis (RDA) carried out on the training-set, which includes the climate variables (MAP, MWP and MSP) and modern pollen assemblages.

**
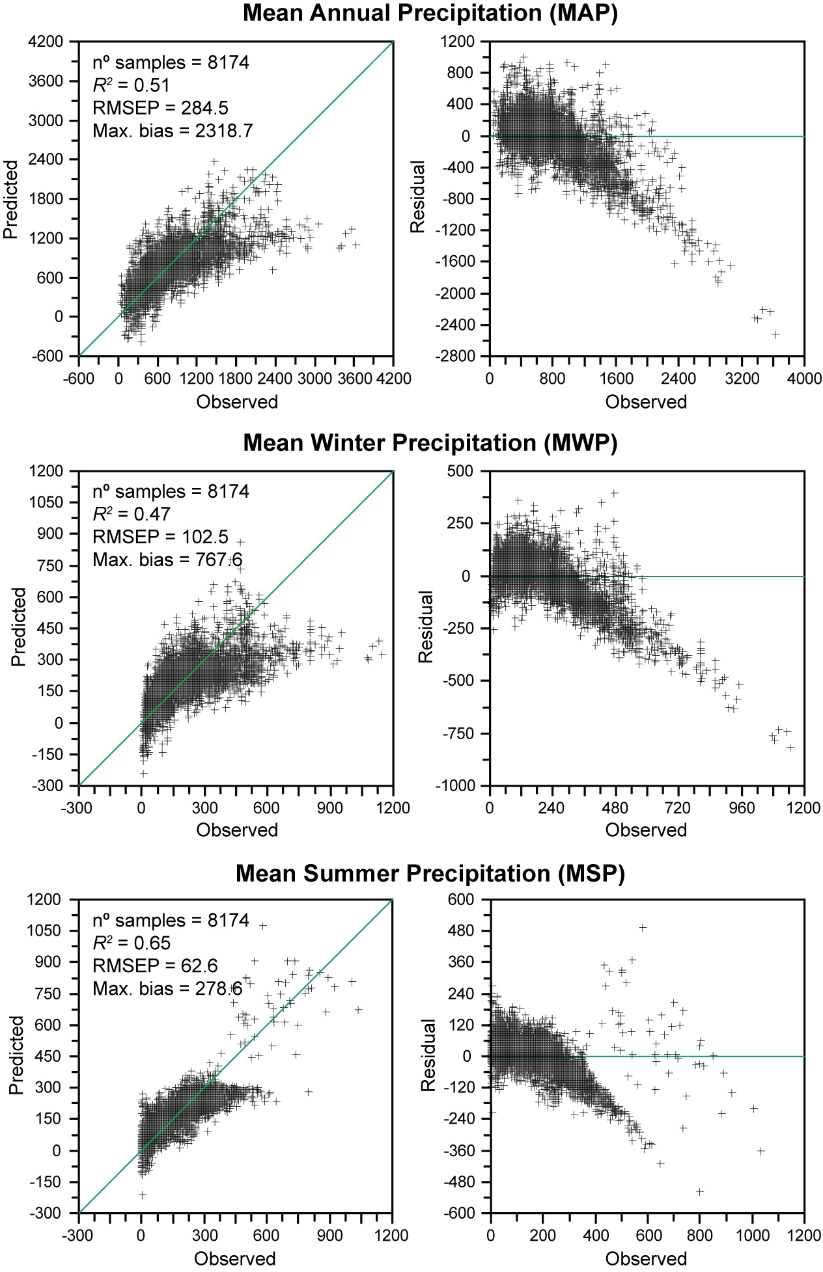
**

**Figure S5.** Scatter plots of the EMPDv2 showing the observed *vs* predicted: mean annual, winter and summer precipitation. The statistical performance of the two-component Weighted Averaging-Partial Least Squares (WA-PLS) method is also shown, including the number of samples (nº samples), coefficient of determination (*R^2^*), root-mean square error of prediction (RMSEP) and maximum bias (Max. bias).


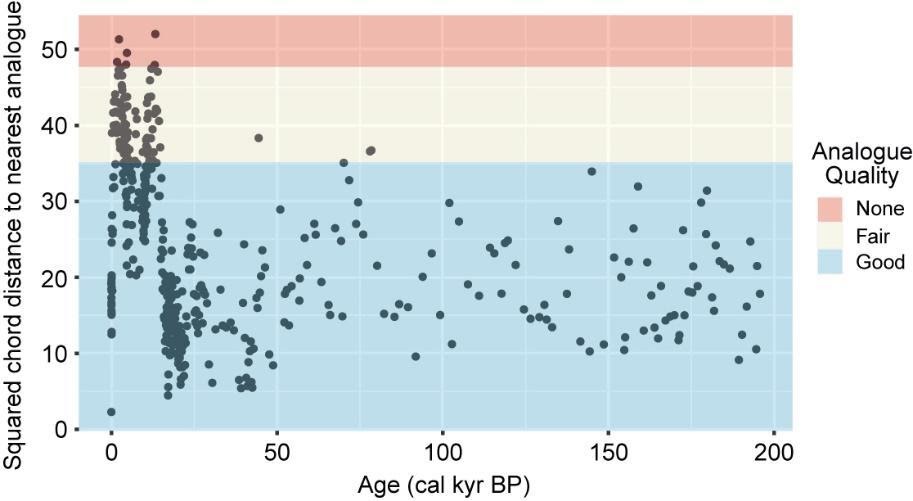


**Figure S6.** Analogue quality for the Padul mean annual precipitation (MAP) reconstruction based on the goodness-of-fit analysis, using the EMPDv2 from Davis et al. (2020)^1^. Blue, yellow and red shadings indicate good-, fair- and non-analogues, respectively.

**
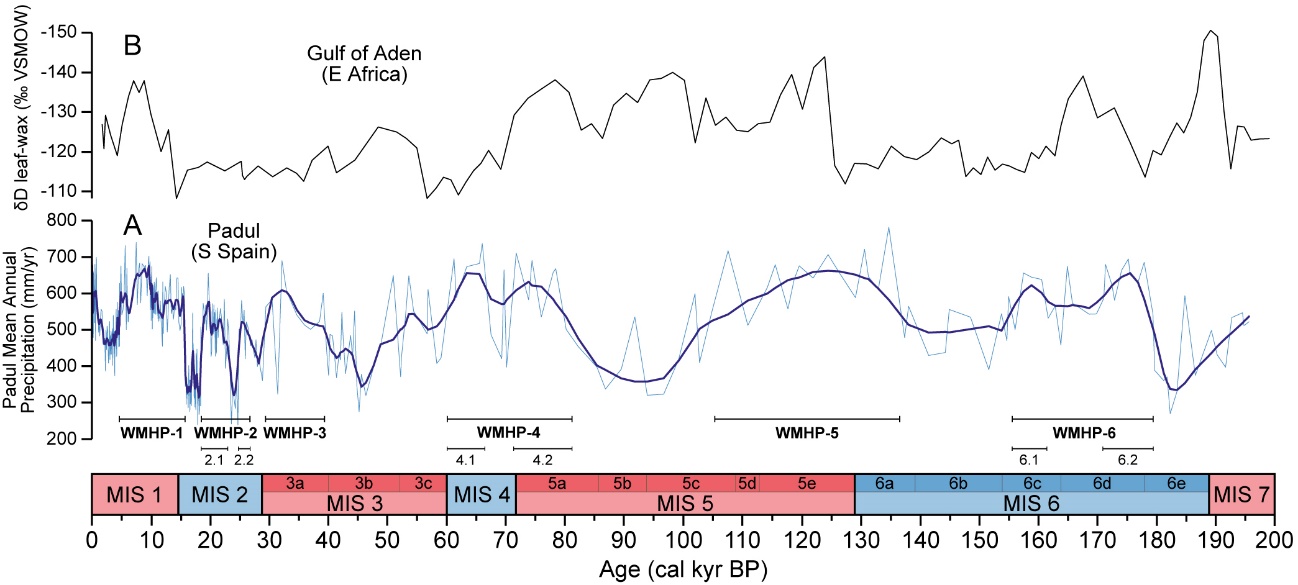
**

**Figure S7.** **(A)** The reconstructed MAP from Padul (LOESS smoothing, span 0.02) with the WMHPs (black horizontal lines) and **(B)** the δD leaf-wax biomarker (ice-volume corrected, ‰) from the gulf of Aden (marine RC09-166 record, horn of Africa)^3^.

**SUPPLEMENTARY** **REFERENCES**

1 Davis, B. A. S. *et al.* The Eurasian Modern Pollen Database (EMPD), version 2. *Earth System Science Data* **12**, 2423-2445, <https://doi.org/10.5194/essd-12-2423-2020> (2020).

2 Camuera, J. *et al.* Vegetation and climate changes during the last two glacial-interglacial cycles in the western Mediterranean: A new long pollen record from Padul (southern Iberian Peninsula). *Quaternary Science Reviews* **205**, 86-105, <https://doi.org/10.1016/j.quascirev.2018.12.013> (2019).

3 Tierney, J. E., deMenocal, P. B. & Zander, P. D. A climatic context for the out-of-Africa migration. *Geology* **45**, 1023-1026, <https://doi.org/10.1130/G39457.1> (2017).
